# Supplementary material for: Evaluation of outpatient treatment for non-hospitalised patients with COVID-19: The experience of a regional centre in the UK
Source: PLoS One. 2023 Mar 15;18(3):e0281915. doi: 10.1371/journal.pone.0281915 (PMC10016683; doi:10.1371/journal.pone.0281915)
Supplement: S1 Table — (DOCX) [file pone.0281915.s001.docx]

**S1 Table: Demographic and clinical details of patients that received Paxlovid**

|  | **Paxlovid (n=5)** |
| --- | --- |
| Median age (range) | 47 (42-59) |
| Female (%) | 4 (80%) |
| **Ethnicity** | |
| White British | 4 (80%) |
| Unknown | 1 (20%) |
| **Indications** | |
| Active solid organ or metastatic cancer | 1 (20%) |
| Chemotherapy meeting criteria in policy | 1 (20%) |
| HIV with CD4 > 350 cells/mm^3^ and additional risk factors | 1 (20%) |
| Haematological malignancy | 1 (20%) |
| Multiple sclerosis | 1 (20%) |
| **Outcomes** | |
| Hospital admission within 28 days | 0 |
| Mortality within 28 days | 0 |
