# Supplementary material for: Evaluation of outpatient treatment for non-hospitalised patients with COVID-19: The experience of a regional centre in the UK
Source: PLoS One. 2023 Mar 15;18(3):e0281915. doi: 10.1371/journal.pone.0281915 (PMC10016683; doi:10.1371/journal.pone.0281915)
Supplement: Attachment — Submitted filename: Response to reviewers.docx [file pone.0281915.s002.docx]

15^th^ December 2022

**Re: Response to reviewers – PONE-D-22-19820**

Dear Dr Lee and the editorial board of PLOS-ONE,

Many thanks for your feedback on our manuscript. Please find attached a revised manuscript addressing each of the reviewer and editor comments, and details of how we have responded to these comments below.

**Journal Requirements:**

1) We have reformatted our manuscript to meet PLOS ONE’s style requirements
2) We have uploaded correspondence from the research governance team in the Research and Innovation department of Nottingham University Hospitals confirming that ethical review was not required for this work.
3) The “funding information” section on the submission portal has been updated with award numbers. Please could the funding statement be recorded as below:

“ATG holds an NIHR-funded Academic Clinical Lecturer post. IPH holds an NIHR Senior Investigator Award (award number NF-SI-0617-10096). The funders had no role in study design, data collection and analysis, decision to publish, or preparation of the manuscript.”

Please note, the ACL post held by ATG is not associated with an award number, therefore this is not stated.

4) The “funding information” section of the manuscript has been removed
5) The “competing interests” section has been updated to confirm that our declarations of interest do not alter our adherence to  PLOS ONE policies on sharing data and materials. This statement should now read as below:

“Competing interests: ATG and JST - None to declare. IPH has a funded research collaboration with Boehringer Ingelheim (unrelated to the topic of this paper). This does not alter our adherence to  PLOS ONE policies on sharing data and materials.”

6) We have reviewed the reference list, which is complete and correct. Reference 1 has been edited slightly to ensure that the reported number of COVID-19 related deaths is up to date (edited from 6.2 to 6.6 million deaths worldwide in the opening paragraph of the introduction). The URLs in references 8, 10, and 15 (original numbers) have been updated and we confirm that these led to the correct information on 1^st^ December 2022. References 11-13 (original numbers) have been updated with the full citations (i.e. page numbers); this information was unavailable at the time of the original submission. We have also added an additional reference to address Reviewer 2’s comment on the deprivation data.

**Reviewer comments**

**Reviewer 1**

1) **Reviewer comment:** “ This very low rate of suitable patients for assessment uncovers criticisms on referral pathway. This point has been underlined by the authors but some points need to be clarified: as for M&M it could be useful to clarify in the last paragraph of "Study design" if the process is computerized, if being within a high risk group is an information currently available for all the patients or physicians have to retrive this info case-by-case and which is the timing for registration/transfer of info to CMDU and so on”

**Response:** We agree that our study reveals inefficiencies in the referral pathway to CMDU. We have added more detail about the CMDU referral pathways to the “study design” section of the revised manuscript. Additions have also been made to the first paragraph of the discussion to expand upon possible reasons for the inefficiencies that we observed.

2) **Reviewer comment:** “Finally, due to the poor external validity of these results I suggest to modify the title in this way: "Evaluation of outpatient treatment for non-hospitalised patients with COVID-19: a mono-institutional experience in UK"”

**Response:** We agree with this comment, and have edited our title to “Evaluation of outpatient treatment for non-hospitalised patients with COVID-19: The experience of a regional centre in the UK”. We have opted to call this a “regional” study rather than mono-institutional, as our CMDU covers a large region of mid- and south Nottinghamshire in the UK, which encompasses areas covered by several different NHS hospital trusts.

**Reviewer 2**

1) **Reviewer comment:** “Resolving symptoms is an exclusion criteria. 266 were excluded with resolving symptoms, given the subjective nature of this, 2 deaths in the resolving symptoms group, and 15 admissions at d28 for COVID suggests this should not be an exclusion criteria for treatment. This could be made clearer”

**Response:** Thank you for this comment. We have added a section to the discussion (3^rd^ paragraph of discussion) to address this. We have referred to the 7 covid-related hospital attendances rather than the 15 hospital admissions within 28 days, as some of these admissions were deemed to be unrelated to Covid-19.

2) **Reviewer comment:** “The fact that those from more deprived backgrounds are also less likely to receive treatment is important but well highlighted. Can the authors give any further insight why?”

**Response:** Unfortunately, the reasons that patients gave for declining treatment were not frequently recorded, and thus we could not analyse this in more detail. We suspect that patients from more deprived backgrounds are less likely to be able to attend for a sotrovimab infusion (for example time off work or childcare), are more reluctant to travel for treatment, or they may be more likely to minimise their symptoms and decline treatment. Furthermore, patients from more deprived backgrounds may be less trusting of and therefore less willing to accept these relatively new treatments. The reasons behind inequitable access to treatment are an important area of further research, and the Nuffield Trust has reported that patients from areas of greater deprivation are more likely to receive poorer quality care across a range of healthcare services (Scobie S, Morris J. Quality and inequality: digging deeper. Nuffield Trust and the Health Foundation. 2020. <https://www.nuffieldtrust.org.uk/public/files/2020-01/quality_inequality>.) We have edited paragraph 6 of the discussion to emphasise this further, and added the reference to the study by Scobie et al.

3) **Reviewer comment:** “Admission at d28 was 3% in untreated vs 1.2% in treated with an OR 0.4 (95%CI 0.12 to 1.36). Can the authors calculate/estimate the sample size they would need to see an effect with this event rate?”

**Response:** Many thanks for this suggestion. On our power calculations, we estimate that, assuming equal matching in the groups and power set at 80%, the required number of subjects to detect a significant difference in outcome based on the event rates we observed would be 1990. We have added this to the manuscript discussion.

4) “Table 2 in vaccination status, would be helpful to add categories for more than x2 and more than x3 vaccinations with percentages”

We have edited Table 2 to include the percentages of patients who had received 1, 2, 3, or 4 doses of a Covid-19 vaccination, and separate section for those that received two doses or less / 3 doses or more of a Covid-19 vaccination.

Many thanks for your continued consideration of our manuscript for publication.

Dr Amanda Goodwin

Dr Jonathan Thompson

Professor Ian Hall
